# Olanzapine as a prophylactic antiemetic for preventing postoperative nausea and vomiting after general anesthesia: A systematic review and meta-analysis

**DOI:** 10.1016/j.clinsp.2024.100345

**Published:** 2024-03-20

**Authors:** Thiago Ramos Grigio, Hans Timmerman, Angela Maria Sousa, André Paul Wolff

**Affiliations:** a Department of Anaesthesiology, Pain Center, University of Groningen, University Medical Center Groningen, Groningen, The Netherlands; b Postgraduate Program of Anaesthesiology, Surgical Sciences and Perioperative Medicine, Faculdade de Medicina da Universidade de São Paulo (USP), São Paulo, SP, Brazil

**Keywords:** Antipsychotic agent, Perioperative care, Postoperative emesis, Postoperative nausea and vomiting, Review, Surgical

## Abstract

**Background:**

The antiemetic effectiveness of olanzapine, as a prophylactic off-label antiemetic drug, for Postoperative Nausea and Vomiting (PONV) is unknown. In this systematic review and meta-analysis, the authors evaluate the efficacy and side effects of olanzapine as a prophylactic antiemetic in adult patients who undergo general anesthesia and assess adverse effects.

**Methods:**

A systematic search was done on electronic bibliographic databases in July 2023. Randomized controlled trials of olanzapine as a prophylactic antiemetic for PONV in adults who underwent general anesthesia were included. The authors excluded non-RCTs and retracted studies. The authors set no date of publication or language limits. The outcomes were the incidence of PONV within 24 h postoperatively and the safety of olanzapine. The risk of bias was assessed according to the tool suggested by the National Heart, Lung, and Blood Institute.

**Results:**

Meta-analysis included 446 adult patients. Olanzapine reduced on average 38 % the incidence of PONV. The estimated risk ratio (95 % CI) of olanzapine versus control was 0.62 (0.42–0.90), *p* = 0.010, I^2^ = 67 %. In the subgroup meta-analysis, doses of olanzapine (10 mg) reduced on average 49 % of the incidence of PONV (RR = 0.51 [0.34–0.77], *p* = 0.001, I^2^ = 31 %).

**Conclusions:**

This systematic review with meta-analysis indicated that olanzapine as a prophylactic antiemetic alone or combined with other antiemetic agents reduced the incidence of postoperative nausea and vomiting. However, this conclusion must be presented with some degree of uncertainty due to the small number of studies included. There was a lack of any evidence to draw conclusions on side effects.

## Highlights

• Olanzapine is identified as a potentially effective prophylactic antiemetic.
• Olanzapine reduces the incidence of postoperative nausea and vomiting.
• Olanzapine has few side effects.

## Introduction

Nausea and vomiting are common postoperative complications in patients who receive general anesthesia and are considered one of the most unwanted symptoms after surgery.^1^ It limits the patient's recovery by reducing appetite, causing sleep disturbances, and delaying physical therapy mobilization.^2^ Other consequences are dehydration, altered electrolytes, and pulmonary aspiration of gastric contents.^3^ Moreover, it might cause a delay in hospital discharge^4^ or raise the hospital readmission rate, which would increase the cost of hospitalization.^5^

Postoperative Nausea and Vomiting (PONV) prophylaxis is performed based on the number of risk factors identified. Risk factors for PONV are diverse and can be divided into patient-related, surgery or anesthesia-related, and postoperative factors.^6^ According to Apfel and colleagues^7^ the four main risk factors are female, postoperative opioid use, non-smoking, and motion sickness or history of PONV in previous surgeries.

Since 2013, recommendations have been published to guide anaesthesiologists in recognizing the risk factors and indicating appropriate prophylaxis.^6^^,^^8^, ^9^, ^10^, ^11^, ^12^, ^13^ The greater the number of risk factors, the greater the number of antiemetics that should be used as prophylaxis. Forty-four drugs can be used as antiemetics.^14^ Despite the guidelines and numerous antiemetics available, the incidence of PONV is still high in certain groups of patients.^15^ Therefore, some medications that have no formal indication as antiemetic drugs are emerging, but there are no studies evaluating the evidence for use as antiemetics or side-effects of these drugs in the postoperative context.

Olanzapine is an atypical antipsychotic drug approved by the U.S. Food and Drug Administration (FDA) for treating schizophrenia, mania, and bipolar disorder. More recently, researchers have shown interest in olanzapine as an antiemetic for PONV and Chemotherapy-Induced Nausea and Vomiting (CINV) due to its broad spectrum of action at receptors related to the pathophysiology of nausea and vomiting.^16^^,^^17^ Olanzapine is usually used as an add-on drug to established antiemetics and its use as a sole antiemetic is limited. In addition, the use of olanzapine for PONV and CINV is off-label. Olanzapine binds to serotoninergic, dopaminergic, muscarinic, and histaminergic receptors. Compared to other antiemetics, olanzapine does not only act on four different receptor types but also on different subtypes within the same receptor type.^18^ Most antiemetics available only bind one type and one receptor subtype responsible for the pathophysiology of nausea and vomiting.^6^ In 2020, an observational study concluded that chronic use of atypical antipsychotics, including olanzapine, is associated with a lower risk of antiemetic administration in the post-anesthesia recovery room.^17^ Conventional antiemetics have good efficacy in reducing the incidence of nausea and vomiting after surgery. However, in patients at high risk of PONV, or with any contraindications to the available antiemetics, and in surgeries at risk for an increased incidence of nausea and vomiting postoperatively, there is still a need to add new medications with antiemetic properties. To date, no systematic review has evaluated the use of olanzapine for PONV prophylaxis.

### Aims of the study

This systematic review and meta-analysis of randomized controlled trials aimed to evaluate the efficacy and safety of prophylactic olanzapine as an antiemetic in adult patients who underwent general anesthesia.

## Methods

### Protocol and registration

This study design is a systematic review and meta-analysis of randomized controlled trials. The protocol is written following the Preferred Reporting Items for Systematic Reviews (PRISMA statement)^19^ and has been registered prospectively at PROSPERO under number CRD42023258420. No adjustments were made after the study commencement. Ethical approval was not required for this study.

### Study search strategy

A literature search was performed using an electronic search by a medical librarian and the first author (TRG). The detailed search strategy is described in Appendix A. Databases that were used: MEDLINE (via PubMed); EMBASE (via Elsevier); Web of Science; Cochrane Central Register of Controlled Trials (CENTRAL); World Health Organization (WHO) International Clinical Trials Registry Platform (ICTRP); Clinical Trials Results; SCIELO; Grey Literature Report.

Reference lists in identified studies were also reviewed for additional studies. The search was performed in March 2023 and updated in July 2023. The duplicates were excluded in Endnote.

### Eligibility criteria

The eligibility criteria followed the components of PICOT design (population, intervention, control, outcome and time):

### Type of studies

Only Randomized Controlled Trials (RCTs) were included, with simple, blocked, or stratified randomization. RCTs that compared olanzapine alone or combined with other antiemetics for PONV against a placebo, another antiemetic drug, or a combination of other antiemetic drugs were included. Non-RCTs and retracted studies were not included. The authors set no date of publication or language limits to obtain the broadest range of studies.

#### Type of participants

The authors considered all human studies that included adult participants (at least 18 years old) who underwent any surgical procedure under general anesthesia with or without combined techniques (local, regional or neuroaxis anesthesia). There were no restrictions on the inclusion of articles related to the type of surgery, type of anesthesia (as long as the patients received general anesthesia), and drugs used intraoperatively. The authors excluded articles with a pediatric population (< 18 years old) and surgical procedures that were performed under only local or regional anesthesia or sedation.

#### Type of intervention

Interventions of interest were prophylactic olanzapine administered preoperatively or intraoperatively to prevent PONV, compared to a placebo or another antiemetic treatment. Olanzapine alone or combined with another antiemetic was compared to placebo or any combination of antiemetics as long as olanzapine was not included.

All doses were analyzed together, and subgroup analyses were done based on different doses of olanzapine.

#### Type of outcome measures

The primary outcome of this study was the incidence of PONV from 0 to 24 h postoperatively. Although the definition of nausea and vomiting may be similar across studies, some authors distinguished the separate incidence of nausea and vomiting, and others characterized them together (e.g., nausea and/or vomiting; nausea and vomiting). In this study, the authors considered any described incidence of the outcomes. The data were analyzed as a single outcome independent of how it was described. Outcome data could be either the primary or secondary outcome of the studies.

The secondary outcome of this study was the safety of olanzapine. The authors considered the presence or absence of the following adverse events: skin rashes, drowsiness, dry mouth, sedation, sleepiness, cardiac arrhythmias, drowsiness, dizziness, extrapyramidal reactions, akathisia, akinesia, dyskinesia, fatigue, pruritus, and events otherwise reported.

#### Time of outcome assessment

Patients who were evaluated at least 24 h after surgery were included in this study. The primary and secondary outcomes were assessed from 0 to 24 h after surgery.

### Study selection

The Rayyan platform (Rayyan, Doha, Qatar)^20^ was used to manage the selection process by two authors independently (TRG and HT). It was divided into a two-stage process. First step: two authors (TRG and HT) independently assessed all titles and abstracts retrieved by the search strategies. Articles that met the inclusion and avoided the exclusion criteria were marked as ‘potentially eligible’. Second step: two authors (TRG and HT) independently read the full text of the ‘potentially eligible’ articles to confirm their eligibility. If there were disagreements between authors, a third reviewer (APW) was consulted to reach a consensus.

### Data collection process

The procedures for data extraction were done by one reviewer (TRG) and for accuracy checked by a second reviewer (HT). A pre-established data extraction sheet was used. An email was sent to the authors to request raw data to check the results.

### Data items

The following data were collected: name of the first author, year of publication, country of origin, sample size, details on participants, age, sex, type of surgery, intervention, comparison, time of measurement of the outcome, anesthesia induction, anesthesia maintenance, and outcomes of interest.

Nausea was defined as an unpleasant sensation of having the urge to vomit. Vomit was described as a physical event as a forceful expulsion of gastric contents through the mouth. Retching was considered when the content of the gastrointestinal tract was forced into the esophagus without expulsion of the vomitus.^21^

### Study risk of bias assessment

The quality of eligible trials was assessed according to the tool suggested by the National Heart, Lung, and Blood Institute (NHLBI) .^22^ The tool (Appendix B) is composed of 14 questions of quality assessment. It includes questions about description as randomized trial (item 1), allocation concealment (items 2 and 3), blinding (items 4 and 5), the similarity of groups at baseline (item 6), dropout (items 7 and 8), adherence (item 9), avoidance of other interventions (item 10) outcome measures assessment (item 11), power calculation (item 12), prespecified outcomes (item 13), and intention-to-treat analysis (item 14).

Before using this tool, two articles on a different topic were randomly selected to assess the level of understanding of the questions. Each question was discussed, and it was verified that both authors (TRG and HT) had the same understanding of the meaning of the questions as stated in this scale.

The risk of bias was assessed independently by two review authors (TRG and HT), and disagreements were solved by a third reviewer (APW). Each question was graded as ‘yes’, ‘no,’ or ‘unclear’/‘not reported’/‘not applicable’. These answers reflect a low risk of bias, high risk of bias, and uncertain bias, respectively. A low risk of bias translates to a rating of good quality, and a high risk of bias translates to a rating of poor quality. The NHLBI tool considers a ‘fatal flaw’ a study with high dropout rates, high differential dropout rates, no ITT analysis, or other unsuitable statistical analysis (e.g., completers-only analysis). The authors created a table with the reviewers’ answers for each of the 14 questions.

### Unity of analysis

#### Analysis of demographics

The demographic variables were analyzed: age and sex.

#### Heterogeneity assessment

Statistical heterogeneity was considered by means of the Chi-Square test (*p <* 0.05 as significance cut-off) and I^2^ test (I^2^ < 40 %, 40 %‒60 %, and > 60 % represent low, moderate, and high heterogeneity, respectively among RCTs). If possible, in an attempt to explain some of the observed heterogeneity, the authors tried to do a subgroups analysis with different doses of olanzapine, type of anesthesia (total intravenous anesthesia, inhalational anesthesia), type of surgery (laparoscopic surgery, gynecological surgery, cholecystectomy), and combination prophylaxis (olanzapine plus standard treatment) or monotherapy (olanzapine alone).

#### Measures of treatment effect and analysis procedures

For dichotomous variables, risk ratios were used. Mean differences were calculated for continuous variables. A 95 % Confidence Interval was used. Random-effects meta-analysis was performed considering the heterogeneity and the availability of data. The authors used the Review Manager 5.4.1 software (The Cochrane Collaboration, London, United Kingdom) .^23^

## Results

### Study selection

**Fig. 1:**
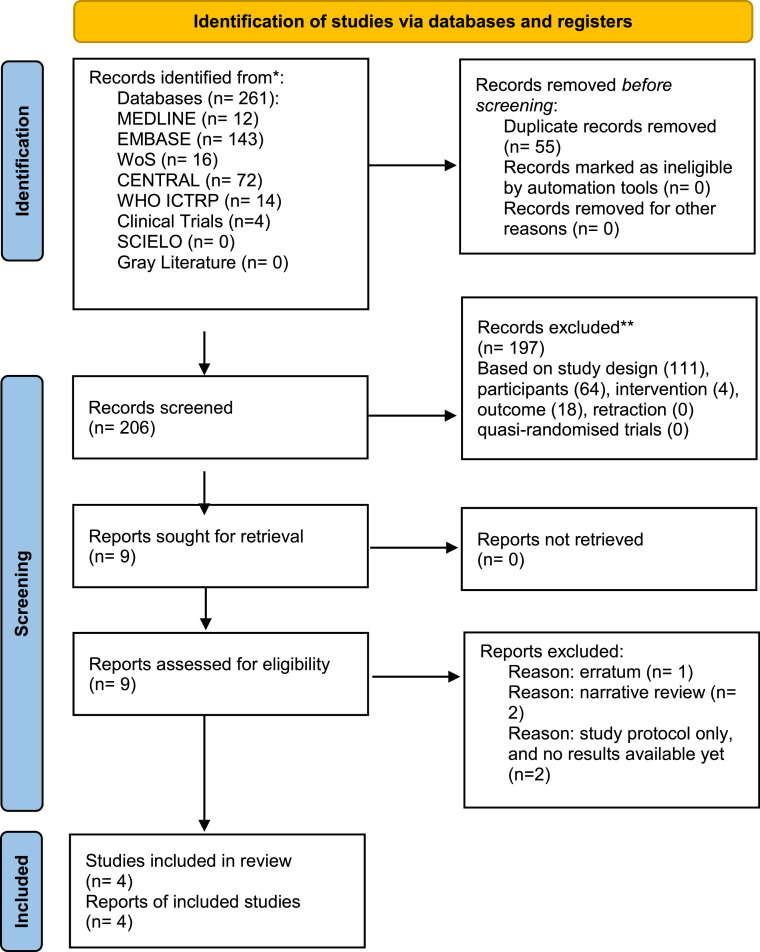
PRISMA 2020 flow diagram for new systematic reviews. WHO ICTRP, World Health Organization International Clinical Trials Registry Platform (ICTRP); WoS, Web of Science.

The search strategy (Appendix A) identified 261 manuscripts, and 55 duplicates were removed, leaving 206 studies to be screened against title and abstract. Agreement on screening abstract was 100 % between authors (TRG and HT), and Cohen's kappa 1.00. Of these, 198 studies were excluded. The study selection procedure flow diagram presents the reasons for excluding the article (Fig. 1).

Nine studies were assessed for full-text eligibility. There was no disagreement among the authors regarding the inclusion criteria. Two full-text narrative reviews^24^^,^^25^ two studies protocol without results, and one erratum^26^ on the paper by Hyman et al. .^27^ were excluded.

Overall, four randomized controlled trials^27^, ^28^, ^29^, ^30^ were eligible for systematic review, including 508 adult patients (263 patients in the olanzapine group and 245 patients in the control group). One trial was published in 2013^28^ and the others in respectively 2020^27^ 2022^29^ and 2023.^30^ Three trials were published in English^27^^,^^28^^,^^30^ and one manuscript was written in Japanese.^29^ This article^29^ came after identifying the study registry at the WHO ICTRP website. The authors were contacted, and they sent the full published text written in Japanese, which was translated afterward. In addition, an email was sent to all authors to request the raw data to check accuracy. To check the accuracy of the results published, the authors sent an e-mail to the authors of all the studies requesting the raw data. Only the raw data from two studies were re-analyzed for statistics.^27^^,^^30^ Seki and colleagues^29^ did not send the data but answered the questions regarding the lack of information in the statistical analysis of the opioid use data. The authors did not receive an answer from one group even after several attempts.^28^

### Study characteristics

The studies have been summarized in Table 1. The mean population's age (±SD) was 40.8 (± 9.0) years. Most participants were women (99 %). ASA's physical health status was reported in one study.^28^

**Table 1:** Summary of studies.

**Study**	**Year**	**Country**	**Sample size**	**Patients**	**Age (Mean)**	**Female Gender**	**Surgery**	**Number of groups**	**Interventions**	**Comparisons**	**Time of measurement**	**Type of anesthesia**	**Induction**	**Maintenance**	**Results**^a^	**Measured outcome**	**Adverse events reported**
Grigio, T.R. et al.	2023	Brazil	96	Adult with oncological disease, age 18‒60y, high risk for PONV (3 or 4 risk factors), previous history of CINV	44.2	96.8 %	Medium or major surgery	2	Olanzapine 10 mg + dexamethasone 4 mg + ondansetron 4 mg	Dexamethasone 4 mg + ondansetron 4 mg	0‒6 h, 0‒24 h and 24‒48 h after surgery	General anesthesia + neuraxial anesthesia	Propofol	TIVA + fentanyl	↓ incidence of PONV (60 % less)	Postoperative nausea or vomiting (or retching), only nausea, only vomiting	Lightheadedness/dizziness, dry mouth, itching, sleepness, hypotension, headache, restless, anxiety, sleep disturbance
Hyman, J.B. et al.	2020	USA	140	Adult female, age 18‒50y	37	100 %	Ambulatory gynecologic or plastic surgery	2	Olanzapine 10 mg + dexamethasone 8 mg + ondansetron 4 mg	Dexamethasone 8 mg + ondansetron 4 mg	‒24 h postdischarge, 0‒24 h after surgery	General anesthesia	Midazolam 2 mg IV + Propofol 1.5 to 2.5 mg/kg + duccinylcholine 1 to 2 mg/kg or rocuronium 0.6 mg/kg	Sevoflurane + fentanyl	↓ incidence of PONV (39 % less)	Postoperative nausea and/or vomiting	Sedation, visual disturbance, urinary retension, lightheadedness/dizziness
Seki, H. et al	2022	Japan	210	Adult female, age 20‒65y, at least 2 factors for PONV	41.4	100 %	Laparoscopic gynecological surgery	2	Olanzapine 5 mg + dexamethasone 6.6 mg	Dexamethasone 6.6 mg	0.5‒2 h, 2‒6 h, 6‒24 h after surgery	General anesthesia	?	Fentanyl + remifentanyl	↓ incidence of PONV (21 % less)	Postoperative nausea, vomiting and use of metoclopramide	Hypotension, headache
Ibrahim, M. et al	2013	Egypt	82	Adult female, ASA I or II	42	100 %	Breast surgery (mastectomy, tumor biopsy, reductive mastoplasty and other plastic surgery)	3	Olanzapine 5 mg and olanzapine 10 mg	Ondansetron 16 mg orally and placebo	0‒2 h, 2‒6 h, 6‒12 h and 12‒24 h after surgery	General anesthesia	Midazolam1–2 mg + propofol 2‒3 mg/kg + rocuronium 1 mg/kg	Sevoflurane + fentanyl 2‒5 mcg/kg + nitrous oxide	NA	Complete response (without nausea, and vomiting, no rescue therapy), “no nausea” and “no emesis/’	Headache, sedation, anxiety, restlessness, and abnormal muscle movements

a Only significant between-group differences (*p <* 0.05) are shown; ↑, Significantly increased; ↓, Significantly decreased; NA, Not Applicable.

The authors of two studies considered Apfel's risk factors for the inclusion of participants.^29^^,^^30^ Among these, one study^30^ included patients with three or four risk factors according to Apfel, and one study^29^ included patients with at least two risk factors according to Apfel.

Three studies^27^^,^^28^^,^^30^ were conducted at a single center (Brazil, Egypt, or USA), and one study^29^ was run in a multi-center setting in Japan. No studies were funded by industry.

Active interventions consisted of olanzapine as a single prophylactic agent^28^ or olanzapine in combination with one (dexamethasone)[29] or two other antiemetics (dexamethasone, ondansetron) .^27^^,^^30^ The control arm of the studies consisted of a placebo^28^ one antiemetic (dexamethasone), or a combination of two antiemetics (dexamethasone and ondansetron) .^27^^,^^30^ One study investigated four groups^28^ and the other studies investigated two groups.^27^^,^^29^^,^^30^

Olanzapine was administered orally at 5 mg^28^^,^^29^ or10 mg^27^^,^^28^^,^^30^ before surgery. The other antiemetics were administered intraoperatively in all studies.

The studies included had different characteristics of type of surgery, and type of anesthesia, although all patients received general anesthesia. Except for one study^30^ that assessed patients who underwent general anesthesia in conjunction with neuraxial anesthesia, in the other three studies^27^, ^28^, ^29^ only general anesthesia was applied. One study was conducted on ambulatory gynecological or plastic surgeries^27^ one study on laparoscopic gynecological surgery^29^ one study on breast surgery^28^ and one study evaluated medium and major surgeries (radical mastectomies with or without flap rotation, expander exchanges, myocutaneous flaps, gastroduodenopancreatectomy, abdominoperineal resection; gynecological cytoreduction with or without cystectomy, hemipelvectomy, peritonectomy; hip arthroplasty, femur endoprosthesis surgery; pulmonary lobectomy, pulmonary metastasectomy, mediastinal tumor resection) .^30^

Maintenance of anesthesia was done with sevoflurane in three studies^27^, ^28^, ^29^ and one study used total venous anesthesia.^30^ There was no significant difference between groups regarding the total use of intraoperative opioids in all studies.^27^, ^28^, ^29^, ^30^ All studies reported postoperative opioid use; there was no statistically significant difference between groups in opioids administered postoperatively in two studies^28^^,^^30^ except for a difference between groups in opioid administration in the Post-anesthesia Care Unit (PACU) despite randomization in two studies.^27^^,^^29^ In both studies, the control group had a statistically significant higher use of opioids in the PACU.

### Primary outcome

The authors of all the studies reported the incidence of nausea and vomiting in different ways. One study^27^ reported the incidence of nausea and/or vomiting together, one study^30^ reported the incidence of nausea or vomiting (or retching) together, and one study^29^ reported the incidence of nausea, vomiting, and use of metoclopramide together. Three studies^27^^,^^29^^,^^30^ reported the outcomes from 0 and 24 hours after surgery. The remaining study^28^ reported the incidence of “no nausea” and “no vomiting” separately from 0‒2 h and 2‒24 h, and there was no report of the incidence of the outcomes from 0 h to 24 h after surgery. An email was sent to the authors^28^ to request the incidence of nausea and vomiting together or separately from 0h to 24 h or the raw data, but the authors did not receive any feedback.

### Secondary outcome

Safety outcomes were reported in the studies within the first 24 hours after surgery, but there was variability in the type of endpoint assessed between the studies. One study^27^ showed a higher incidence of sedation with olanzapine use. In the three other studies^28^, ^29^, ^30^ there was no difference between the side effects of treatment and the control group.

### Risk of bias

The risk of bias is described in Table 2. The raters (TRG and HT) agreed on 84 % of the items scored. Disagreements between assessors were resolved by discussion. After discussion, a 100 % agreement was reached.

**Table 2:** Quality assessment of controlled interventions studies.

**Author Year**	**Hyman 2020**	**Ibrahim 2013**	**Seki 2022**	**Grigio 2023**
**Criteria**				
1. Was the study described as a randomized, a randomized trial, a randomized clinical trial, or an RCT?	Yes	Yes	Yes	Yes
2. Was the method of randomization adequate (i.e., use of randomly generated assignment)?	NR	CD	Yes	Yes
3. Was the treatment allocation concealed (so that assignments could not be predicted)?	CD	NR	Yes	Yes
4. Were study participants and providers blinded to treatment group assignment?	Yes	Yes	Yes	Yes
5. Were the people assessing the outcomes blinded to the participants' group assignments?	Yes	No	Yes	Yes
6. Were the groups similar at baseline on important characteristics that could affect outcomes (e.g., demographics, risk factors, co-morbid conditions)?	Yes	Yes	Yes	Yes
7. Was the overall drop-out rate from the study at endpoint 20 % or lower of the number allocated to treatment?	Yes	Yes	Yes	Yes
8. Was the differential drop-out rate (between treatment groups) at endpoint 15 percentage points or lower?	Yes	Yes	Yes	Yes
9. Was there high adherence to the intervention protocols for each treatment group?	Yes	Yes	Yes	Yes
10. Were other interventions avoided or similar in the groups (e.g., similar background treatments)?	Yes	Yes	Yes	Yes
11. Were outcomes assessed using valid and reliable measures, implemented consistently across all study participants?	Yes	Yes	Yes	Yes
12. Did the authors report that the sample size was sufficiently large to be able to detect a difference in the main outcome between groups with at least 80 % power?	Yes	Yes	Yes	Yes
13. Were outcomes reported or subgroups analyzed pre-specified (i.e., identified before analyses were conducted)?	Yes	Yes	Yes	Yes
14. Were all randomized participants analyzed in the group to which they were originally assigned, i.e., did they use an intention-to-treat analysis?	Yes	Yes	No	Yes

CD, Cannot Determine; NA, Not Applicable; NR, Not Reported.

All studies were described as randomized clinical trials. The randomization method of the two studies was not reported.^27^^,^^28^ Allocation concealment could not be determined in one study.^28^ People assessing the outcomes were not blinded to the participants’ group assignments in one study.^28^ All studies^27^, ^28^, ^29^, ^30^ had similar groups at baseline characteristics. The overall drop-out rates and differential drop-out rates were low between all treatment groups. Adherence was high to the intervention protocols for each treatment group. The presence/absence of PONV and side effects were self-reported by the patients in all studies. All the authors reported that the sample size was calculated with at least 80 % power. In one study^29^ not all the randomized participants were analyzed (no Intention-To-Treat [ITT] analysis was done); therefore this study has poor quality.

### Meta-analysis primary outcome

**Fig. 2:**
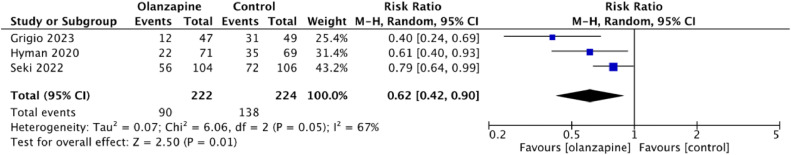
Forest plot showing the incidence of postoperative nausea and vomiting in participants who received olanzapine vs. placebo or active treatment. Pooled risk ratio for incidence of PONV. 95 % CI, 95 % Confidence Interval; M-H, Mantel–Haenszel test; PONV, Postoperative Nausea and Vomiting.

Three studies,^27^^,^^29^^,^^30^ were eligible for the meta-analysis, including 446 adult patients (222 patients in the olanzapine group and 224 patients in the control group). One study^28^ was excluded from the meta-analysis because authors reported only the incidence of “no nausea” and “no emesis” separately in the time frames 0‒2 h and 2‒24 h, and not from 0 h to 24 h. Meta-analysis showed the benefit of prophylactic olanzapine in preventing postoperative nausea and vomiting compared to control: RR = 0.62 (0.42–0.90), *p =* 0.010, I^2^ = 67 % (Fig. 2). There was high heterogeneity within the studies.

**Fig. 3:**
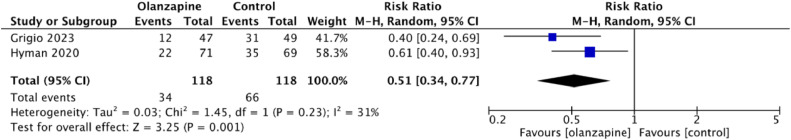
Forest plot showing incidence of postoperative nausea and vomiting in participants who received 10 mg olanzapine vs placebo or active treatment. Pooled risk ratio for incidence of PONV. 95 % CI, 95 % Confidence Interval; M-H, Mantel–Haenszel test; PONV, Postoperative Nausea and Vomiting.

The subgroup analysis was only possible when analyzing different doses of olanzapine (Fig. 3). Only two doses of olanzapine (5 mg and 10 mg) were studied in the included articles, and it was only possible to carry out a subgroup analysis of olanzapine 10 mg since two studies^27^^,^^30^ used this dose. The remaining study^29^ used olanzapine 5 mg, and for this reason, no subgroup analysis was done. In the subgroup analysis, two studies^27^^,^^30^ were included, and one study^29^ with another dose of olanzapine with a high risk of bias was excluded. Therefore, the two studies^27^^,^^30^ with a low risk of bias remained. Doses of olanzapine (10 mg) reduced on average 49 % of the incidence of PONV (RR = 0.51 [0.34–0.77] *p =* 0.001, I^2^ = 31 %). This analysis still showed a benefit of prophylactic olanzapine with dexamethasone and ondansetron compared to control, with low heterogeneity. The authors did not perform other subgroup analyses due to the lack of studies for comparison.

### Meta-analysis secondary outcome

The authors did not perform a meta-analysis of safety as a secondary endpoint because although outcomes were reported in the studies, there was variability in the type of endpoint assessed between the studies. In addition, meta-analysis was not possible regarding the type of anesthesia, type of surgery, and combination prophylaxis because all these variables in the studies were different, and it was not possible to classify them into groups.

## Discussion

### Key observation

This is the first systematic review and meta-analysis of prophylactic olanzapine for postoperative nausea and vomiting in adults who have undergone general anesthesia. The present systematic review and meta-analysis show that using or adding olanzapine to dexamethasone only or dexamethasone plus ondansetron as a prophylactic antiemetic reduces the incidence of PONV more than placebo or dexamethasone and/or ondansetron within 24 hours after surgery. Moreover, in the subgroup analysis, olanzapine 10 mg in combination with dexamethasone and ondansetron statistically significantly reduce the incidence of PONV. No conclusions could be drawn on adverse effects. The results of this study apply to the female population since 99 % of the participants included were women. There was high heterogeneity within the studies in the main analysis. One study,^29^ has a high risk of bias, and therefore its results should be interpreted with caution. The other two studies^27^^,^^30^ have no significant risk of bias after the evaluation of each question on the assessment tool (Table 2). In the subgroup analysis, after the exclusion of the study with a high risk of bias^29^ the two studies^27^^,^^30^ included in this second analysis have low risk of bias and low heterogeneity.

### Clinical implications

According to the meta-analysis, olanzapine as an add-on antiemetic reduces the incidence of postoperative nausea and vomiting by 38 %. This result is higher than found in previous literature that endorses 26 % risk reduction per antiemetic.^31^ However, a combination of five antiemetics has an odds ratio of 0.15 for rescue/self-required PONV occurrences.^32^ This greater reduction in the incidence of nausea and vomiting may be related to the effect of olanzapine combined with only dexamethasone or dexamethasone plus ondansetron. Olanzapine acts in four different receptor types (serotoninergic, dopaminergic, muscarinic, and histaminergic) and subtypes (dopaminergic: D1–D4; serotoninergic: 5-HT2a, 5-HT2c, 5-HT3 and 5-HT6) responsible for the pathophysiology of nausea and vomiting.^18^ The authors speculate that the more antiemetics from different classes are combined, the greater the chance of blocking all PONV pathways. In the present study, it was not possible to evaluate the isolated effect of olanzapine as an antiemetic because no studies evaluated olanzapine as a single agent for PONV prophylaxis in the meta-analysis.

The authors of the four studies included in the systematic review used olanzapine doses of 5 or 10 mg. In the meta-analysis, the dose of olanzapine used in two studies^27^^,^^30^ was 10 mg, and one study, with a high risk of bias, used 5 mg of olanzapine.^29^ Following this, the authors performed a subgroup analysis with olanzapine doses of 10 mg. Doses of 10 mg olanzapine reduced the incidence of PONV by 49 %. After excluding the study with a high risk of bias, there was a greater reduction in the incidence of PONV with 10 mg of olanzapine when compared to the initial analysis.

The studies reported the incidence of adverse effects, however, meta-analysis was not possible due to the great heterogeneity of adverse effects. Therefore, the authors individually evaluated the side effects of each study, and no serious side effects were reported. The side effects are diverse because they may be caused by the antagonism of the four types of receptors (serotoninergic, dopaminergic, muscarinic, and histaminergic) blocked by olanzapine. The most commonly reported side effects of olanzapine are sedation, constipation, headache, tremors, dry mouth, asthenia, dizziness, drowsiness, urinary retention, hypotension, extrapyramidal reaction, hyperkinetic muscle activity, akinesia, drug-induced Parkinson's disease, dyspepsia.^33^ Although there is a wide variety of side effects, only one study^27^ showed that olanzapine has statistically significantly more sedation than ondansetron plus dexamethasone. All three other studies^28^, ^29^, ^30^ found no significant differences when olanzapine was compared to other antiemetics or placebo. Since few studies have been published in the surgical population, future studies must evaluate as many side -effects already reported by olanzapine, not only the most common adverse effects.

There was a significant heterogeneity (I^2^ = 67 %) within the studies (RR = 0.62 [0.42–0.90] *p =* 0.01). However, I^2^ should be interpreted cautiously because as the number of studies is small, I^2^ may overestimate heterogeneity by an average of 12 percentage points.^34^ The authors can explain inconsistencies because different antiemetic doses, antiemetic combinations, types of surgery, types of maintenance anesthesia and group populations were analyzed. Nonetheless, in the subgroup analysis, only two studies,^27^^,^^30^ with a low risk of bias were analyzed. The heterogeneity remarkably reduced (I^2^ = 31 %) and findings remained statistically significant (RR = 0.51 [0.34–0.77] *p =* 0.001). Heterogeneity may have been reduced due to the exclusion of the article with a high risk of bias and the fact that only 10 mg doses of olanzapine were analyzed.

Due to the limited number of studies on olanzapine as a perioperative antiemetic, the authors included a broad subgroup of participants, with different types of surgery, anesthesia and drugs used intraoperatively, leading to great heterogeneity. Therefore, the results of this study should be evaluated with caution in specific groups of patients and surgeries.

There is a significant variety in how the Apfel score was established and used in PONV research. According to Darvall et al. ^35^ 48 % of the studies about PONV measured the four component risk factors, 22 % of the studies reported the calculated Apfel risk scores, and even less, around 5 %, defined each component of the risk factors. There is also significant heterogeneity in the definition of postoperative opioid use. Postoperative opioid use was defined as originally intended (“anticipated use”) in 54 % or “actual” in 18 % of the studies, and it was unclear in 28 %.

Although the Apfel criteria consider postoperative opioid use to be a risk factor, it is not possible to predict this risk at all times before surgery. Depending on some procedures and patient characteristics, it is unpredictable to indicate whether or not the patient will require more potent analgesia (opioids) for pain control. In this way, predicting one of the four risk factors of the Apfel criteria is difficult. In this meta-analysis, although the risk factors according to Apfel's criteria.^31^ were not reported in two papers^29^^,^^30^ all studies showed at least three recognized risk factors, including other known risk factors that are not included in the Apfel criteria (use of inhalation anesthesia, duration of surgery, and type of surgery) .^6^ The female sex was the most studied in the studies since there is a higher incidence of PONV in women.^6^ Therefore, it remains challenging to evaluate the efficacy of any medications for the prophylaxis of PONV in men.

The included articles did not show consensus on how the primary outcome was reported. According to Apfel^36^ nausea and vomiting should be reported and presented separately with the corresponding incidences. Authors of only one study^30^ reported the incidence of nausea and vomiting separately. Other authors reported it as nausea and/or vomiting^27^ together, or nausea, vomiting, and use of metoclopramide together.^29^ Since four articles were included in the systematic review, the authors decided to consider all the outcomes together regardless of how it was reported. However, this may provide non-comparable results. Consequently, there might be an overestimation or underestimation of the 38 % reduction of PONV.

### Strengths and limitations

This is the first systematic review that evaluated olanzapine as prophylaxis for postoperative nausea and vomiting. The authors used the PRISMA checklist as a basis for writing the text. Risk of bias was used as a tool to assess the quality of the studies. Besides, the authors used a wide database to search for articles, and the Japanese article was obtained and translated into English after emailing the authors since it was not indexed in the databases, but the study record was identified. This systematic review indicates the beneficial effect of olanzapine on postoperative nausea and vomiting, mainly in women, since most of the participants included in the studies are female. However, further exploration of olanzapine in perioperative settings is needed.

This systematic review has some limitations. First, only a small number of articles and patients could be included. Second, the articles did not show consensus on how the outcome was reported, and the authors combined the results of all the studies regardless of how they were reported. Third, it was not possible to do a meta-analysis with doses of olanzapine of 5 mg, different types of anesthesia or surgery, and monotherapy with olanzapine due to a lack of studies. Fourth, it was not possible to evaluate the effect of olanzapine according to Apfel's risk factors for PONV. Although the results of this study are intended for patients with any risk of nausea and vomiting after surgery, it was possible to identify that all articles presented at least three risk factors for PONV considering the type of surgery, type of anesthesia, and patient characteristics. Fifth, data collected on the incidence of PONV from the studies were extracted from both primary and secondary outcomes, and raw data from one article was not available. This may be a limitation as the data analyzed were not always extracted from the primary outcomes of the studies. This review is not generalizable to the whole population of patients because women were by far the majority of the population evaluated for the effect of the medicine.

In the primary analysis, one study^29^ has a high risk of bias; therefore its results should be interpreted cautiously. However, in the subgroup analysis that evaluated the effect of olanzapine 10 mg, this study was excluded from the analysis.

The studies analyzed revealed that olanzapine can be used as prophylaxis as an add-on drug for postoperative nausea and vomiting in patients undergoing general anesthesia. However, this conclusion must be presented with some degree of uncertainty due to the small number of studies included and a majority of females. There was a lack of any evidence to draw conclusions on side effects. Further research is required to study the efficacy and safety of olanzapine.

## Conclusion

This systematic review revealed that olanzapine effectively reduces the incidence of PONV in adults who have undergone general anesthesia. The present meta-analysis concludes that prophylactic olanzapine in combination with dexamethasone only or dexamethasone and ondansetron reduces the incidence of postoperative nausea and vomiting within 24 h after surgery compared to the association of dexamethasone and ondansetron or dexamethasone only.

## Prospero registry number

CRD42023258420.

## CRediT authorship contribution statement

**Thiago Ramos Grigio:** Conceptualization, Data curation, Investigation, Methodology, Resources, Visualization, Formal analysis, Writing – original draft. **Hans Timmerman:** Conceptualization, Data curation, Investigation, Methodology, Resources, Visualization, Writing – review & editing. **Angela Maria Sousa:** Conceptualization, Methodology, Project administration, Resources, Supervision, Validation, Writing – review & editing. **André Paul Wolff:** Conceptualization, Methodology, Project administration, Resources, Supervision, Validation, Writing – review & editing.

## Conflicts of interest

The authors declare no conflicts of interest.

## Appendix Supplementary materials

Image, application 1

Image, application 2

## References

[1] Eberhart L.H.J., Morin A.M., Wulf H., Geldner G. (2002). Patient preferences for immediate postoperative recovery. Br J Anaesth.

[2] White P.F., O'Hara J.F., Roberson C.R., Wender R.H., Candiotti K.A. (2008). The impact of current antiemetic practices on patient outcomes: a prospective study on high-risk patients. Anesth Analg.

[3] Taenzer A.H., Havidich J.E., Coté CJ, Lerman J, Anderson BJ (2019). A Practice of Anesthesia For Infants and Children.

[4] Pizzi L.T., Toner R., Foley K. (2012). Relationship between potential opioid-related adverse effects and hospital length of stay in patients receiving opioids after orthopedic surgery. Pharmacotherapy.

[5] Dzwonczyk R., Weaver T.E., Puente E.G., Bergese S.D. (2012). Postoperative nausea and vomiting prophylaxis from an economic point of view. Am J Ther.

[6] Gan T.J., Belani K.G., Bergese S. (2020). Fourth consensus guidelines for the management of postoperative nausea and vomiting. Anesth Analg.

[7] Apfel C.C., Laara E., Koivuranta M., Greim C.A., Roewer N. (1999). A simplified risk score for predicting postoperative nausea and vomiting: conclusions from cross-validations between two centers. Anesthesiology.

[8] McCracken G., Houston P., Lefebvre G. (2008). Guideline for the management of postoperative nausea and vomiting. J Obstet Gynaecol Can.

[9] Gan T.J., Meyer T., Apfel C.C. (2003). Consensus guidelines for managing postoperative nausea and vomiting. Anesth Analg.

[10] Gan T.J., Meyer T.A., Apfel C.C. (2007). Society for ambulatory anesthesia guidelines for the management of postoperative nausea and vomiting. Anesth Analg.

[11] Gan T.J., Diemunsch P., Habib A.S. (2014). Consensus guidelines for the management of postoperative nausea and vomiting. Anesth Analg.

[12] Moningi S., Patki A., Padhy N., Ramachandran G. (2019). Enhanced recovery after surgery: an anesthesiologist's perspective. J Anaesthesiol Clin Pharmacol.

[13] Apfelbaum J.L., Silverstein J.H., Chung F.F. (2013). Practice guidelines for postanesthetic care: an updated report by the American society of anesthesiologists task force on postanesthetic care. AnesthesiologyAnesthesiology.

[14] Weibel S., Rücker G., Eberhart L.H. (2020). Drugs for preventing postoperative nausea and vomiting in adults after general anaesthesia: a network meta-analysis. Cochrane Database Syst Rev.

[15] Grigio T.R., Sousa A.M., Magalhães G.G.N., Ashmawi H.A., Vieira J.E. (2020). Aprepitant plus palonosetron for the prevention of postoperative nausea and vomiting after breast cancer surgery: a double blind, randomized trial. Clinics (Sao Paulo).

[16] Yoodee J., Permsuwan U., Nimworapan M. (2017). Efficacy and safety of olanzapine for the prevention of chemotherapy-induced nausea and vomiting: a systematic review and meta-analysis. Crit Rev Oncol Hematol.

[17] Jabaley C.S., Gray D.W., Budhrani G.S. (2020). Chronic atypical antipsychotic use is associated with reduced need for postoperative nausea and vomiting rescue in the postanesthesia care unit: a propensity-matched retrospective observational study. Anesth Analg.

[18] Davis M.P., Sanger G.J. (2020). The benefits of olanzapine in palliating symptoms. Curr Treat Options Oncol.

[19] Page M.J., McKenzie J.E., Bossuyt P.M. (2021). The prisma 2020 statement: an updated guideline for reporting systematic reviews. Int J Surg.

[20] Ouzzani M., Hammady H., Fedorowicz Z., Elmagarmid A. (2016). Rayyan-a web and mobile app for systematic reviews. Syst Rev.

[21] Zhong W., Shahbaz O., Teskey G. (2021). Mechanisms of nausea and vomiting: current knowledge and recent advances in intracellular emetic signaling systems. Int J Mol Sci.

[22] National Heart, Lung, and BIood institute, https://www.nhlbi.nih.gov/health-topics/study-quality-assessment-tools [accessed 2 September 2023]

[23] (RevMan) RM. Version 5.4 ed: the cochrane collaboration; 2020.

[24] Smith H.S., Laufer A. (2014). Opioid induced nausea and vomiting. Eur J Pharmacol.

[25] Wallenborn J., Eberhart L.H.J., Kranke P. (2009). Does everything remain the same in the pharmacotherapy of postoperative nausea and vomiting?. Anasthesiol Intensivmed Notfallmed Schmerzther.

[26] No authors listed (2020). Erratum: olanzapine for the prevention of postdischarge nausea and vomiting after ambulatory surgery: a randomized controlled trial. Anesthesiology..

[27] Hyman J.B., Park C., Lin H.M. (2020). Olanzapine for the prevention of postdischarge nausea and vomiting after ambulatory surgery: a randomized controlled trial. Anesthesiology.

[28] Ibrahim M., Eldesuky H.I., Ibrahim T.H. (2013). Oral olanzapine versus oral ondansetron for prevention of post-operative nausea and vomiting. a randomized, controlled study. Egypt J Anaesth.

[29] Seki H., Kuratani N., Shiga T. (2022). The prophylactic effect of oral olanzapine on postoperative nausea and vomiting in laparoscopic gynecological surgery: a randomized clinical trial. Masui.

[30] Grigio T.R., Timmerman H., Martins J.V.B., Slullitel A., Wolff A.P., Sousa A.M. (2023). Olanzapine as an add-on, pre-operative anti-emetic drug for postoperative nausea or vomiting: a randomised controlled trial. Anaesthesia.

[31] Apfel C.C., Korttila K., Abdalla M. (2004). A factorial trial of six interventions for the prevention of postoperative nausea and vomiting. N Engl J Med.

[32] Williams B.A., Holder-Murray J.M., Nettrour J.F. (2023). Aim for zero: prevention of postoperative nausea and vomiting using an off-patent five-drug multimodal approach. Br J Anaesth.

[33] Davis M.P., Sanger G.J. (2021). The benefits of olanzapine in palliating symptoms. Curr Treat Options Oncol.

[34] von Hippel P.T. (2015). The heterogeneity statistic I2 can be biased in small meta-analyses. BMC Med Res Methodol.

[35] Darvall J., Handscombe M., Maat B., So K., Suganthirakumar A., Leslie K. (2021). Interpretation of the four risk factors for postoperative nausea and vomiting in the apfel simplified risk score: an analysis of published studies. Can J Anaesth.

[36] Apfel C.C., Roewer N., Korttila K. (2002). How to study postoperative nausea and vomiting. Acta Anaesthesiol Scand.

