# Supplementary material for: Olanzapine as a prophylactic antiemetic for preventing postoperative nausea and vomiting after general anesthesia: A systematic review and meta-analysis
Source: Clinics (Sao Paulo). 2024 Mar 20;79:100345. doi: 10.1016/j.clinsp.2024.100345 (PMC10972821; doi:10.1016/j.clinsp.2024.100345)
Supplement: Supplementary file 1 [file mmc1.docx]

**SISTEMATIC REVIEW SEARCH STRATEGY**

P = postoperative patients

I = olanzapine

C = control (any antiemetic or placebo)

O = nausea and vomiting

Type of study: RCT

No language barriers

**Pubmed:**

("Postoperative Nausea and Vomiting"[Mesh] OR ponv[tiab] OR postoperat*[tiab] OR post-operat*[tiab] OR post-procedur*[tiab] OR postprocedur*[tiab] OR postsurg*[tiab] OR postdischarge[tiab] OR post-discharge[tiab] OR anesth*[tiab] OR anaesth*[tiab] OR postanesth*[tiab] OR postanaesth*[tiab] OR surg*[tiab])

 AND

 ("Nausea"[Mesh] OR "Vomiting"[Mesh] OR vomit*[tiab] OR nause*[tiab] OR sick*[tiab] OR retch*[tiab] OR emes*[tiab])

 AND

 ("Olanzapine"[Mesh] OR olanzapine[tiab] OR 2 methyl 4 4 methyl 1 piperazinyl 10h thieno 2, 3 b 1, 5 benzodiazepine[tiab] OR dopin tab[tiab] OR jolyon md[tiab] OR lanzac[tiab] OR y 170053[tiab] OR ly170053[tiab] OR midax[tiab] OR olace[tiab] OR olan[tiab] OR olansek[tiab] OR relprevv[tiab] OR zalasta[tiab] OR zelta[tiab] OR zypadhera[tiab] OR Zyprexa[tiab] OR Zolafren[tiab] OR LY 170053[tiab])

**Embase:**

('postoperative nausea and vomiting'/exp OR (ponv OR postoperat* OR ‘post-operat*’ OR ‘post-procedur*’ OR postprocedur* OR postsurg* OR postdischarge OR ‘post-discharge*’ OR anesth* OR anaesth* OR postanesth* OR postanaesth* OR surg*):ab,ti,kw)

AND

('nausea'/exp OR 'vomiting'/exp OR (vomit* OR nause* OR sick* OR retch* OR emes*):ab,ti,kw)

AND

('olanzapine'/exp OR (olanzapine OR ‘2 methyl 4 4 methyl 1 piperazinyl 10h thieno 2, 3 b 1, 5 benzodiazepine’ OR ‘dopin tab’ OR ‘jolyon md’ OR lanzac OR ‘y 170053’ OR ly170053 OR midax OR olace OR olan OR olansek OR relprevv OR zalasta OR zelta OR zypadhera OR Zyprexa OR Zolafren OR ‘LY 170053’):ab,ti,kw)

**Web of Science:**

TS= (ponv OR postoperat* OR “post-operat*” OR “post-procedur*” OR postprocedur* OR postsurg* OR postdischarge OR “post-discharge*” OR anesth* OR anaesth* OR postanesth* OR postanaesth* OR surg*)

AND

TS= (vomit* OR nause* OR sick* OR retch* OR emes*)

AND

TS= (olanzapine OR “2 methyl 4 4 methyl 1 piperazinyl 10h thieno 2, 3 b 1, 5 benzodiazepine” OR “dopin tab” OR “jolyon md” OR lanzac OR “y 170053” OR ly170053 OR midax OR olace OR olan OR olansek OR relprevv OR zalasta OR zelta OR zypadhera OR Zyprexa OR Zolafren OR “LY 170053”)

**Cochrane:**

(ponv OR postoperat* OR “post-operat*” OR “post-procedur*” OR postprocedur* OR postsurg* OR postdischarge OR “post-discharge*” OR anesth* OR anaesth* OR postanesth* OR postanaesth* OR surg*)

AND

(vomit* OR nause* OR sick* OR retch* OR emes*)

AND

(olanzapine OR “2 methyl 4 4 methyl 1 piperazinyl 10h thieno 2, 3 b 1, 5 benzodiazepine” OR “dopin tab” OR “jolyon md” OR lanzac OR “y 170053” OR ly170053 OR midax OR olace OR olan OR olansek OR relprevv OR zalasta OR zelta OR zypadhera OR Zyprexa OR Zolafren OR “LY 170053”)

**WHO International Clinical Trials Registry Platform (**[**https://trialsearch.who.int/**](https://trialsearch.who.int/)**)**

("Postoperative Nausea and Vomiting" OR ponv OR postoperat* OR post-operat* OR post-procedur* OR postprocedur* OR postsurg* OR postdischarge OR post-discharge OR anesth* OR anaesth* OR postanesth* OR postanaesth* OR surg*)

 AND

 ("Nausea" OR "Vomiting" OR vomit* OR nause* OR sick* OR retch* OR emes*)

 AND

 ("Olanzapine" OR olanzapine OR “2 methyl 4 4 methyl 1 piperazinyl 10h thieno 2, 3 b 1, 5 benzodiazepine” OR dopin OR jolyon OR lanzac OR “y 170053” OR ly170053 OR midax OR olace OR olan OR olansek OR relprevv OR zalasta OR zelta OR zypadhera OR Zyprexa OR Zolafren OR LY 170053)

**Clinical Trial Results**

("Postoperative Nausea and Vomiting" OR ponv OR postoperat* OR post-operat* OR post-procedur* OR postprocedur* OR postsurg* OR postdischarge OR post-discharge OR anesth* OR anaesth* OR postanesth* OR postanaesth* OR surg*)

 AND

 ("Nausea" OR "Vomiting" OR vomit* OR nause* OR sick* OR retch* OR emes*)

 AND

 ("Olanzapine" OR olanzapine OR “2 methyl 4 4 methyl 1 piperazinyl 10h thieno 2, 3 b 1, 5 benzodiazepine” OR dopin OR jolyon OR lanzac OR “y 170053” OR ly170053 OR midax OR olace OR olan OR olansek OR relprevv OR zalasta OR zelta OR zypadhera OR Zyprexa OR Zolafren OR LY 170053)

**Scielo**

("Postoperative Nausea and Vomiting" OR ponv OR postoperat* OR post-operat* OR post-procedur* OR postprocedur* OR postsurg* OR postdischarge OR post-discharge OR anesth* OR anaesth* OR postanesth* OR postanaesth* OR surg*)

 AND

 ("Nausea" OR "Vomiting" OR vomit* OR nause* OR sick* OR retch* OR emes*)

 AND

 ("Olanzapine" OR olanzapine OR “2 methyl 4 4 methyl 1 piperazinyl 10h thieno 2, 3 b 1, 5 benzodiazepine” OR dopin OR jolyon OR lanzac OR “y 170053” OR ly170053 OR midax OR olace OR olan OR olansek OR relprevv OR zalasta OR zelta OR zypadhera OR Zyprexa OR Zolafren OR LY 170053)
