# Supplementary material for: Olanzapine as a prophylactic antiemetic for preventing postoperative nausea and vomiting after general anesthesia: A systematic review and meta-analysis
Source: Clinics (Sao Paulo). 2024 Mar 20;79:100345. doi: 10.1016/j.clinsp.2024.100345 (PMC10972821; doi:10.1016/j.clinsp.2024.100345)
Supplement: Supplementary file 2 [file mmc2.docx]

| **Quality Assessment of Controlled Intervention Studies** | | | |
| --- | --- | --- | --- |
|  |  |  |  |
| **Criteria** | **Yes** | **No** | **Other (CD, NR, NA)*** |
|  |  |  |  |
| 1. Was the study described as a randomized, a randomized trial, a randomized clinical trial, or an RCT? |  |  |  |
| 2. Was the method of randomization adequate (i.e., use of randomly generated assignment)? |  |  |  |
| 3. Was the treatment allocation concealed (so that assignments could not be predicted)? |  |  |  |
| 4. Were study participants and providers blinded to treatment group assignment? |  |  |  |
| 5. Were the people assessing the outcomes blinded to the participants' group assignments? |  |  |  |
| 6. Were the groups similar at baseline on important characteristics that could affect outcomes (e.g., demographics, risk factors, co-morbid conditions)? |  |  |  |
| 7. Was the overall drop-out rate from the study at endpoint 20% or lower of the number allocated to treatment? |  |  |  |
| 8. Was the differential drop-out rate (between treatment groups) at endpoint 15 percentage points or lower? |  |  |  |
| 9. Was there high adherence to the intervention protocols for each treatment group? |  |  |  |
| 10. Were other interventions avoided or similar in the groups (e.g., similar background treatments)? |  |  |  |
| 11. Were outcomes assessed using valid and reliable measures, implemented consistently across all study participants? |  |  |  |
| 12. Did the authors report that the sample size was sufficiently large to be able to detect a difference in the main outcome between groups with at least 80% power? |  |  |  |
| 13. Were outcomes reported or subgroups analyzed prespecified (i.e., identified before analyses were conducted)? |  |  |  |
| 14. Were all randomized participants analyzed in the group to which they were originally assigned, i.e., did they use an intention-to-treat analysis? |  |  |  |
|  |  |  |  |
| Quality Rating (Good, Fair, or Poor) |  |  |  |
|  |  |  |  |
| Rater #1 initials: |  | | |
| Rater #2 initials: |  | | |
| Additional Comments (If POOR, please state why): |  | | |
|  |  |  |  |
| *CD, cannot determine; NA, not applicable; NR, not reported | |  |  |
